# Genome-wide identification and expression analysis of *DREB* family genes in cotton

**DOI:** 10.1186/s12870-023-04180-4

**Published:** 2023-03-30

**Authors:** Jiuchang Su, Shanglin Song, Yiting Wang, Yunpeng Zeng, Tianyu Dong, Xiaoyang Ge, Hongying Duan

**Affiliations:** 1 College of Life Sciences, Henan Normal University, Xinxiang, 453007 China; 2 State Key Laboratory of Cotton Biology, Institute of Cotton Research, Chinese Academy of Agricultural Sciences, Anyang, 455000 China

**Keywords:** *DREB*, Transcription factors, Phylogenetic analysis, Gene expression, Cotton

## Abstract

**Background:**

Dehydration responsive element-binding (*DREB*) transcription factors are widely present in plants, and involve in signalling transduction, plant growth and development, and stress response. *DREB* genes have been characterized in multiple species. However, only a few *DREB* genes have been studied in cotton, one of the most important fibre crops. Herein, the genome‑wide identification, phylogeny, and expression analysis of *DREB* family genes are performed in diploid and tetraploid cotton species.

**Results:**

In total, 193, 183, 80, and 79 putative genes containing the AP2 domain were identified using bioinformatics approaches in *G. barbadense*, *G. hirsutum*, *G. arboretum*, and *G. raimondii*, respectively. Phylogenetic analysis showed that based on the categorization of *Arabidopsis DREB* genes, 535 *DREB* genes were divided into six subgroups (A1–A6) by using MEGA 7.0. The identified *DREB* genes were distributed unevenly across 13/26 chromosomes of A and/or D genomes. Synteny and collinearity analysis confirmed that during the evolution, the whole genome duplications, segmental duplications, and/or tandem duplications occurred in cotton *DREB* genes, and then *DREB* gene family was further expanded. Further, the evolutionary trees with conserved motifs, *cis*-acting elements, and gene structure of cotton *DREB* gene family were predicted, and these results suggested that *DREB* genes might be involved in the hormone and abiotic stresses responses. The subcellular localization showed that in four cotton species, DREB proteins were predominantly located in the nucleus. Further, the analysis of *DREB* gene expression was carried out by real-time quantitative PCR, confirming that the identified *DREB* genes of cotton were involved in response to early salinity and osmotic stress.

**Conclusions:**

Collectively, our results presented a comprehensive and systematic understanding in the evolution of cotton *DREB* genes, and demonstrated the potential roles of *DREB* family genes in stress and hormone response.

**Supplementary Information:**

The online version contains supplementary material available at 10.1186/s12870-023-04180-4.

## Background

With the global climatic change, the plant growth and development were challenged with variable abiotic stimuli, ultimately resulting in massive agricultural losses [1, 2], and this problem continues to worsen. To respond to various stresses, plants have gradually evolved a series of sophisticated regulatory mechanisms [2, 3], in which the stress-responsive transcriptional factors and hormonal signaling transduction events function together to form an interconnected network [3, 4]. Transcription factors are involved in activating or repressing the rate of transcription of their target gene(s) [5, 6]. Previous reports confirmed that the numerous stress-responsive transcription factors in plants, such as *WRKY* [7], *MYB* [8], and *AP2/ERF* [9], were induced under abiotic stimuli. According to the number of domains and their specific binding sequences, *AP2/ERF* family were classified into the four major groups: *AP2* subfamily, *ERF* subfamily, *DREB* subfamily, and *RAV* subfamily [10, 11].

The distribution of *DREB* genes were varied greatly in plant kingdom. Based on their structural characteristics, *DREB* genes could be further divided into six subgroups: A1–A6 [12]. *DREB* transcription factors are one of the most promising regulons for abiotic stress tolerance in plants that directly bound to six nucleotides (A/GCCGAC) of DRE [13, 14]. These sequences could be found in the promoter regions of drought and cold responsive genes [15–18]. *DREB* subfamily could regulate genes involved in diverse biological processes [19], such as growth, development, and stress responses, through transcriptional and post-translational modification. For example, the overexpression of soybean *DREB1* could improve the drought stress tolerance of transgenic wheat in the field [20]. Recently, a study demonstrated the important role of *DREB*s not only in stress response, but in development and photosynthesis of *Saccharum spontaneum* [21]. In mung bean, 30 *DREB* genes have been identified and characterized, five of genes are significantly upregulated under drought condition [22]. In barley, 41 *DREB* genes have been discovered, among which, many genes are highly expressed in response to drought and salt stimuli [23]. Additionally, a large amount of *DREB* genes have been identified in wheat genome, and the overexpression of *TaDREB3* could confer the plant tolerance to heat, dehydration, and salt stresses [24]. A number of *DREB* genes have been identified and characterized in a wide variety of plant species [25–29], in which the function of *DREB* families has been extensively reviewed. Collectively, the ample reports confirmed that under the abiotic and biotic stresses, *ERF* family/*DREB* subfamily genes have emerged as key regulators in plants [30].

Cotton (*Gossypium* spp.) is one of the most important economically crop plants worldwide, and it provides the natural textile fiber and edible oil [31]. Apart from its economic value, cotton is also an excellent model polyploid crop for studying polyploidization, cell elongation, and cell wall biosynthesis [32–34]. Previous reports have been proposed that all diploid cotton species may have evolved from a common ancestor, followed by subsequently diversifying to produce eight groups, including groups A–G and K3 [35, 36]. *G. raimondii* is a wild species, and the draft genome was drawn in detail in previous study [32], in which a voucher specimen of this material has been deposited in a publicly available herbarium. Since the release of whole-genome sequences of the diploid cotton *G. raimondii* [35] and the allotetraploid cotton *G. hirsutum* (TM-1) [37], the genome-wide analysis of *DREB* genes is possible, and will help to elucidate their regulatory functions in plant growth, development and, in particular, stimuli responses. Although some members of *AP2/EREBP* gene families have been studied, the *DREB* superfamily genes remains largely unexplored in cottons.

To fill this gap, we provide a detailed overview of genome-wide analysis in four cotton species, including two diploid species (*G. arboreum* and *G. raimondii*) and two tetraploid species (*G. barbadense* and *G. hirsutum*). Herein, the Cotton Functional Genomics Database was used for systematically analyzing these *DREB* genes, in which the variation in *DREB* family were identified and annotated. Next, we performed bioinformatics analysis for classification of *DREB* genes into six subgroups. Further, the presence of chromosomal distribution, gene duplication events, characteristic conserved motifs, putative *cis*‑acting elements, and gene structure were carried out. The subcellular localization of all DREB proteins were predicted. Finally, the expression changes of several *DREB* gene in *G. hirsutum* were checked during early osmotic and salt stress. In summary, we performed the genome-wide identification and expression analysis of *DREB* family genes in cotton, and verified the roles of *DREB* in abiotic stimuli responses. These findings will also provide useful insights for future studies on functional characterization of *DREB* genes in cotton.

## Results

### Identification and phylogenetic analysis of *DREB* subfamily genes in cotton

**Fig. 1:**
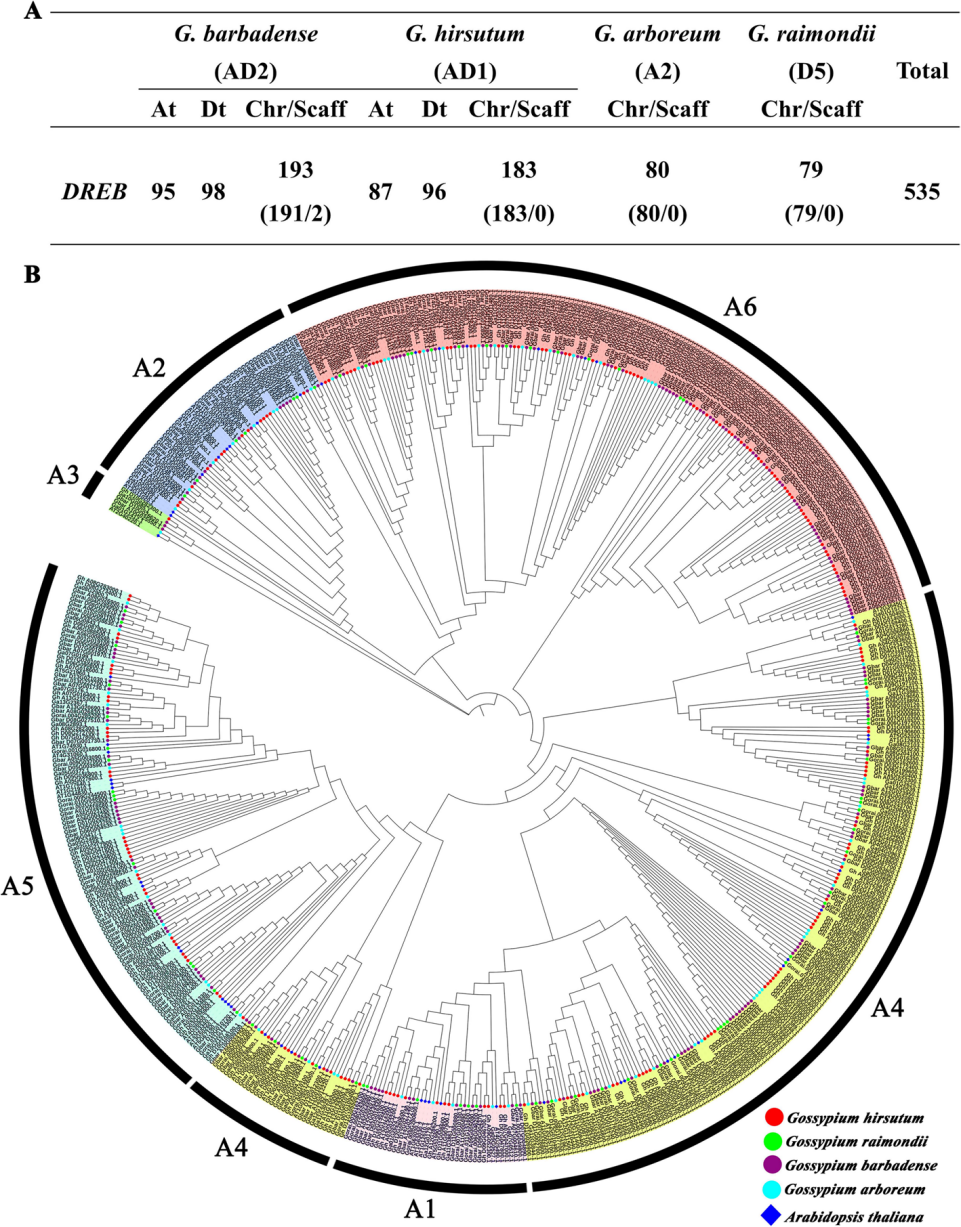
Number of *DREB* genes from four cotton species and a phylogenetic tree of DREB proteins among *Gossypium* spp. and *Arabidopsis thaliana*. **A** The number of *DREB* genes in *G. barbadense*, *G. hirsutum*,* G. arboreum*, and *G. raimondii*, among which, Chr and Scaff indicated respectively the chromosome and scaffold. **B** The unrooted phylogenetic tree was structured with the maximum likelihood method (1000 bootstraps) based on the amino acid sequence of DREB proteins

Herein, the total of 193, 183, 80, and 79 *DREB* genes in *Gossypium barbadense* (AD2), *Gossypium hirsutum* (AD1), *Gossypium arboreum* (A2), and *Gossypium raimondii* (D5) were identified, respectively (Fig. 1A). In *G. barbadense*, 95 *DREB* genes were discovered on At sub-genome, and 98 *DREB* genes were identified on Dt sub-genome. In *G. hirsutum*, 87 were observed in At sub-genome, and 96 were identified in Dt sub-genome. In *Gossypium arboretum* and *Gossypium raimondii*, 80 and 79 *DREB* genes were identified, respectively, on their respective chromosome. According to their species association, all 535 *DREB* genes from four cottons were renamed. Next, the phylogenetic analysis of all putative *DREB* genes from the four *Gossypium* species and 51 *DREB* genes of *A. thaliana* were carried out, based on amino acid sequences of identified DREB proteins by using maximum likelihood method. Based on the subgroup (s) categories from *A. thaliana*, 535 *DREB* genes of four cotton species were divided into six subgroups (Groups A1–A6; Fig. 1B), in which A4 and A3 are the largest and smallest subgroup with 187 and 5 cotton *DREB* genes, respectively. In addition, A1, A2, A5, and A6 subgroup contains 36, 43, 105, and 159 cotton *DREB* genes, respectively. Above results suggested that *DREB* genes of four cotton species and *A. thaliana* were unevenly distributed in all subgroup.

### Chromosomal mapping and gene duplication of *DREB* family genes in cotton

To investigative the genomic distribution of *DREB* genes on cotton chromosome, the physical positions of all *DREB* genes from *G. barbadense*, *G. hirsutum*, *G. arboreum*, and* G. raimondii* was identified by using the position data of *DREB* genes obtained from the Cotton Functional Genomics Database. Meanwhile, the gene duplication events were analyzed for whole genome duplications (WGD), segmental duplications, and/or tandem duplications to investigate the expansion pattern of *DREB* gene family from four cotton species.

**Fig. 2:**
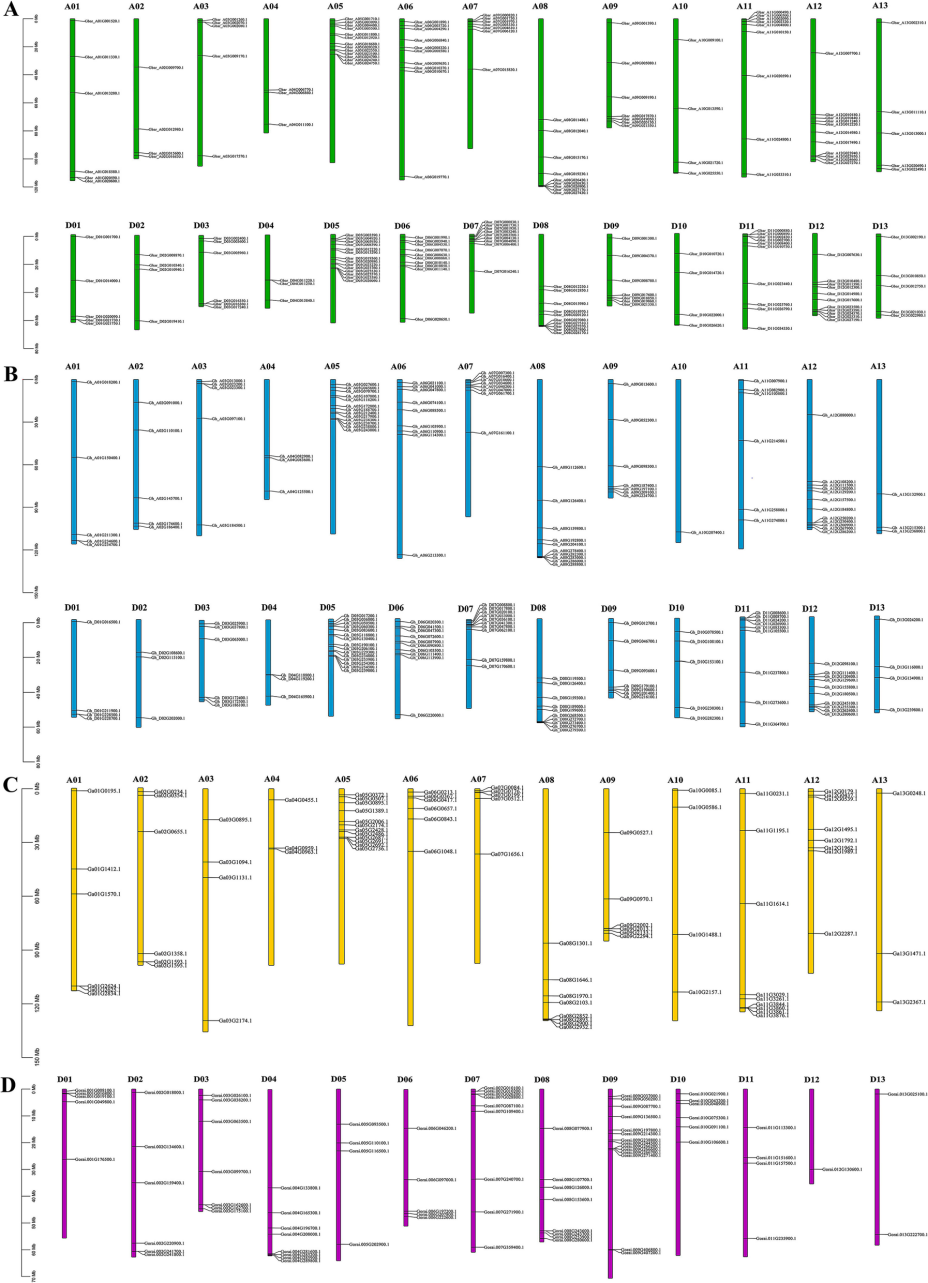
Chromosomal distribution of *DREB* family genes in cotton genome. The chromosome distribution of *DREB* genes from **A** *G. barbadense*, **B** *G. hirsutum*, **C** *G. arboretum*, and **D** *G. raimondii* were shown on their respective chromosomes. The physical positions of *DREB* were mapped according to cotton genome. The scale for the length of chromosome is mega bases (Mb). The chromosome number is shown at the top of each chromosome

**Fig. 3:**
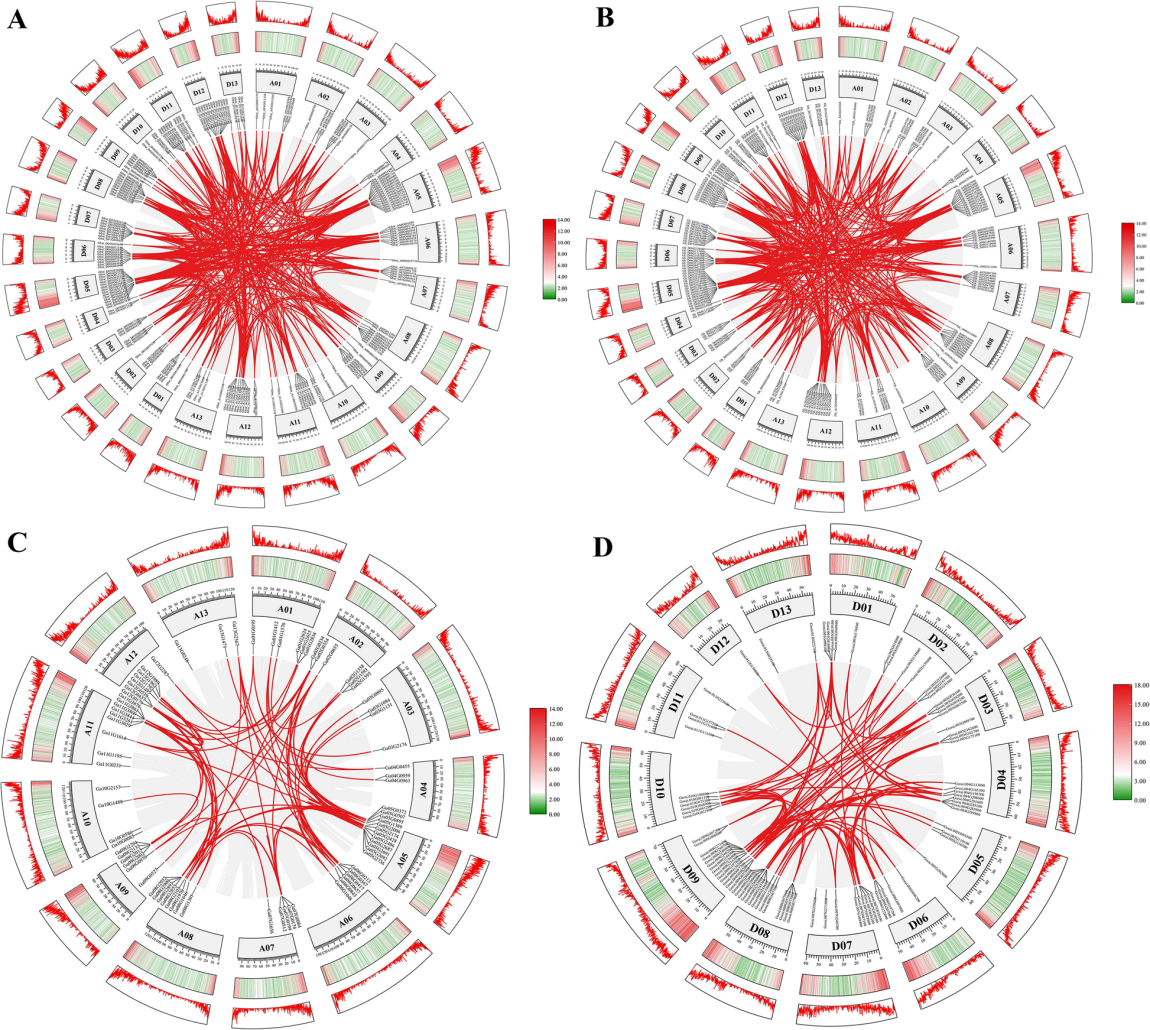
Schematic representation of the duplication of *DREB* genes in the cotton genome. The gene duplication events from **A** *G. barbadense*, **B** *G. hirsutum*, **C** *G. arboretum*, and **D** *G. raimondii* were exhibited on their respective chromosomes. The number of each chromosome is indicated inside each bar. The scale on the above of box is in mega bases (Mb). *DREB* gene pairs with a syntenic relationship are linked by red lines

In *G. barbadense*, 191 *DREB* genes were unevenly distributed on all 26 chromosomes, among which, 93 and 98 genes were located in At and Dt sub-genome, respectively (Fig. 2A). Meanwhile, *Gbar_A07G025290.1* and *Gbar_A08G027720.1* were located in scaffold 278 and scaffold 3, respectively. Our results further showed that in At sub-genome of *G. barbadense*, 6, 4, 5, 3, 13, 10, 7, 9, 7, 4, 9, 11, and 5 *DREB* genes were located on A01 to A13 chromosomes, and A04 and A05 chromosomes contained the minimum and highest number *DREB* genes (3 and 13), respectively (Fig. 2A). Similarly, 5, 4, 6, 3, 14, 10, 9, 10, 7, 4, 10, 11, and 5 *DREB* genes in Dt sub-genome of *G. barbadense* were distributed on D01 to D13 chromosomes, in which chromosome D05 contained the greatest number of *DREB* genes (14), and chromosomes D04 have the lowest number (3) (Fig. 2A). To further investigate the expansion pattern of *DREB* genes in *G. barbadense*, the circos analysis was carried out (Fig. 3A). Our results showed that in 193 *DREB* genes of *G. barbadense*, 188 WGD or segmental duplications were found, and 2 tandem duplications were detected (Fig. 3A, Additional file 1). Furthermore, these genes above were located unevenly on all 26 chromosomes.

In* G. hirsutum*, 183 *DREB* genes were scattered on all 26 chromosomes of At and Dt sub-genomes (Fig. 2B). 87 *DREB* genes were distributed in At sub-genomes, and the number of *DREB* genes from chromosome A01 to A13 was 5, 5, 5, 3, 13, 9, 8, 10, 7, 1, 6, 12, and 3, respectively, in which A05 chromosome and A10 chromosome have maximum and minimum number, respectively (Fig. 2B). Additionally, 96 *DREB* genes were located on Dt sub-genomes. Among these 96 genes, 4, 3, 6, 3, 15, 10, 10, 10, 7, 5, 9, 10, and 4 *DREB* genes were located in D01 to D13 sub-genome, respectively, in which D05 chromosome contained the greatest number of *DREB* genes, and both D02 and D04 chromosomes had the lowest number. Among 183 *DREB* family genes from *G. hirsutum*, 178 genes have WGD or segmental duplications, and 1 gene has tandem duplication (Fig. 3B, Additional file 1). In addition, these 179 genes were located unevenly on all 26 chromosomes.

In* G. arboreum*, 80 *DREB* genes were located unequally on 13 chromosomes. As illustrated in Fig. 2C, our results confirmed that 6, 6, 4, 3, 12, 6, 5, 8, 6, 4, 9, 8, and 3 *DREB* genes were located on chromosomes A1 to A13, respectively. The chromosomes A05 contained the highest number (12) of *DREB* genes. Both A04 and A13 chromosome exhibited the lowest number (3). For 80 *DREB* genes of *G. arboreum*, 67 *DREB* genes showed the WGD or segmental duplications, and 3 tandem genes were observed (Fig. 3C, Additional file 1). Above 70 genes were scattered unevenly on all 13 chromosomes.

In *G. raimondii*, 79 *DREB* genes were unevenly localized on all 13 chromosomes (Fig. 2D). The number of *DREB* genes from D01 to D13 chromosome was 5, 6 7, 8, 4, 5, 9, 8, 16, 6, 4, 1, and 2, respectively, among which, D09 and D12 chromosome contained the highest (16) and minimum (1) number of *DREB* genes, respectively (Fig. 2D). Further, our results confirmed that in all *DREB* genes of *G. raimondii*, 65 WGD or segmental duplications were found, and 3 tandem duplications were showed (Fig. 3D, Additional file 1). These 68 genes were located in all 13 chromosomes.

**Fig. 4:**
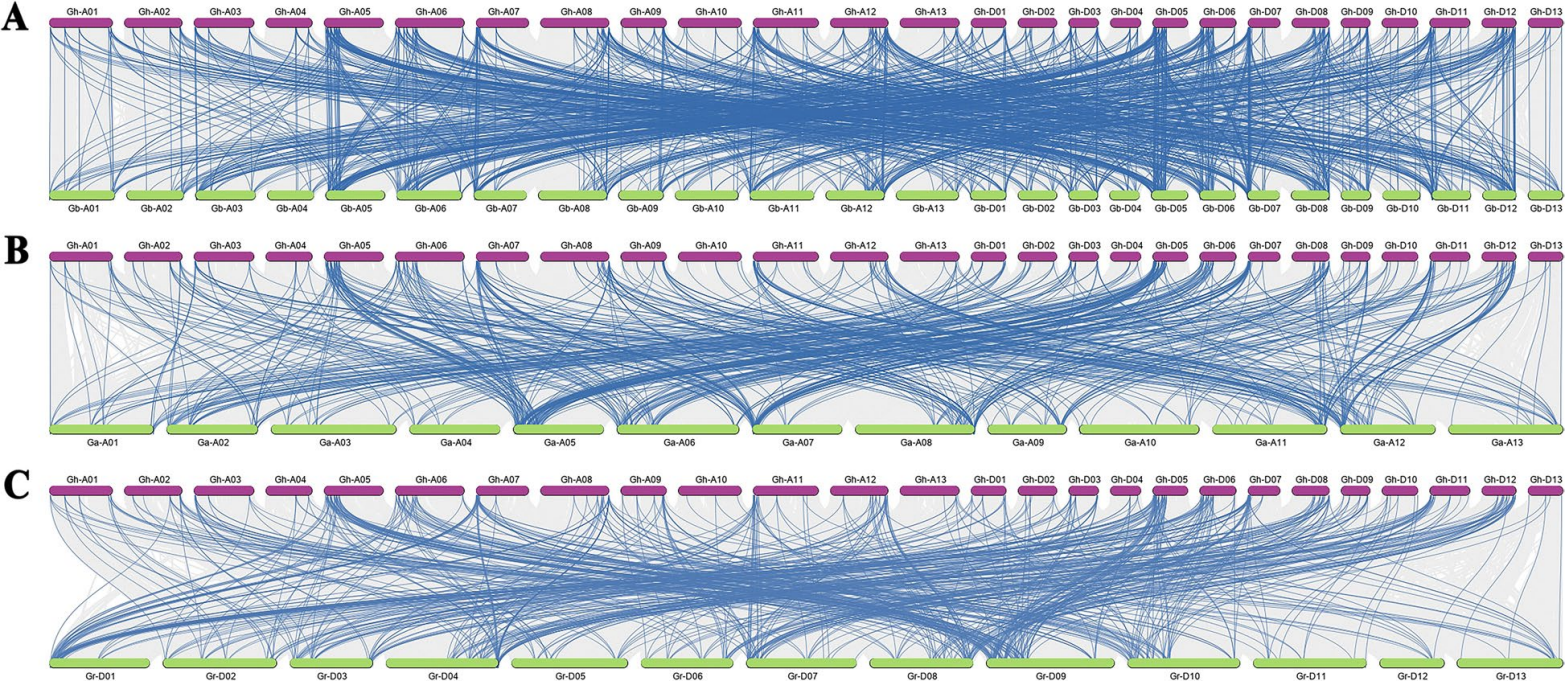
Synteny analysis of *DREB* family genes. Orthologous relationships of *DREB* genes were investigated between **A** *G. hirsutum* and *G. barbadense*; **B** *G. hirsutum* and *G. arboretum*; **C** *G. hirsutum* and *G. raimondii*. The blue lines highlighted collinear *DREB* gene pairs, and the gray lines in the background indicated all collinear blocks

It is known that the *G. hirsutum* and *G. barbadense* are thought to have evolved from the hybridization of A-subgenome of *G. herbaceum* or *G. arboreum* and D-subgenome of *G. raimondii*. To comprehend the evolutionary internal relevancies of *DREB* family genes, the collinearity relationships were identified between *DREB* genes of *G. hirsutum* and related genes from other cotton, including *G. barbadense*, *G. arboreum*, and *G. raimondii* (Fig. 4). MCScanX analysis demonstrated that 181 *DREB* genes of *G. hirsutum* showed collinearity relationships with 195 genes of *G. barbadense* (Fig. 4A, Additional file 2). Meanwhile, these gene loci above in both *G. hirsutum* and *G. barbadense* were located unevenly at At and Dt sub-genomes. There is such relationship between 178 *DREB* genes of *G. hirsutum* and 97 genes of *G. arboretum* (Fig. 4B, Additional file 2). In addition, the collinearity relationships were recognized between 176 genes of *G. hirsutum* and 96 genes of *G. raimondii* (Fig. 4C, Additional file 2). It is worth noting that the number of orthologous gene pairs was 930 between *G. hirsutum* and *G. barbadense*; 551 between *G. hirsutum* and *G. arboretum*; 573 between *G. hirsutum* and *G. raimondii* (Fig. 4, Additional file 2).

### Phylogenetic relationships, conserved motif composition, and *cis*-acting elements, gene structure analysis of *DREB* genes

**Fig. 5:**
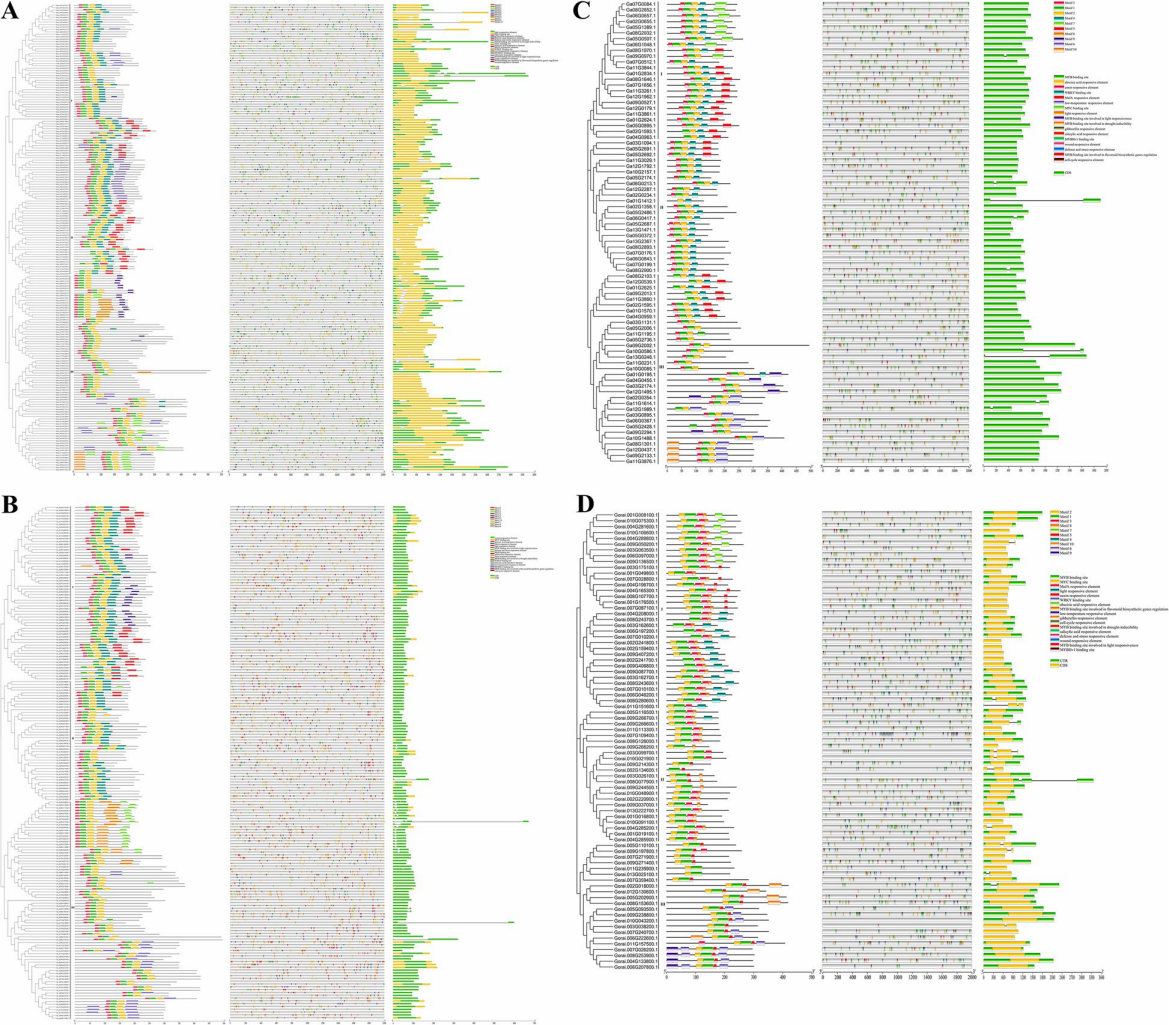
Phylogenetic relationships, protein motif analysis, *cis*-acting element, and gene structure of *DREB* genes in **A** *G. barbadense*, **B** *G. hirsutum*, **C** *G. arboretum*, and **D** *G. raimondii*. The phylogenetic tree was constructed with maximum likelihood method, and DREB proteins were classified into three subgroups (I-III) for each species. Conserved motifs of DREB proteins were identified, and 10 predicted motifs were represented by distinct colored boxes, in which the black lines indicated non-conserved regions. Seventeen *cis*-acting elements were detected in each species, in which they are represented by distinct colored boxes. Gene structure was analyzed, and green and orange boxes represented untranslated region (UTR) and coding sequence (CDS)

To better understand the evolution and structural diversity of *DREB* gene family, the evolutionary relationships with conserved motif, promoter elements, and gene structure were evaluated in *G. barbadense*, *G. hirsutum*, *G. arboreum*, and *G. raimondii*, respectively. In terms of similarity, a total of 10 conserved motifs, motif 1–10, were observed in *DREB* gene family of each species, in which the motif 1 was conserved across all *DREB* genes of four cotton species (Fig. 5). The analysis of promoter *cis*-acting elements showed that among four cotton species, these elements of *DREB* genes have not significant difference (Fig. 5), and in each species, all *DREB* gene families contain some common elements. In addition, unlike *G. arboretum*, both untranslated region (UTR) and coding sequence (CDS) were observed in *DREB* genes of *G. barbadense*, *G. hirsutum*, and *G. raimondii* (Fig. 5). Additionally, AP2 domain was identified in all *DREB* genes among four cotton species (Additional file 3).

For each species, the related information above also reported. According to evolution analysis, firstly, all 193 *DREB* genes of *G. barbadense* were categorized into three groups, I-III (Fig. 5A). Both I and III contained 81 *DREB* genes, and in each group, the motif 1, 2, and 3 were conserved. In group II, the motif 1, 2, and 4 were conserved in 31 *DREB* genes. For 193 *DREB* genes in *G. barbadense*, their gene structure showed significant difference. 35.75% of genes were UTR-less, and both UTR and CDS were observed in 64.24% of genes. 23.32% of genes have introns, and 76.68% genes have no introns. Next, the evolutionary tree classified 183 *DREB* genes of *G. hirsutum* into 3 clades, including I (62), II (43), and III (78) group (Fig. 5B). In group I, motif 1, 2, and 4 were conserved. Both motif 1 and 3 were identified in group II and III, respectively. For 183 *DREB* genes in *G. hirsutum*, 80.32% of genes were UTR-less, and 19.67% of genes have both UTR and CDS. Meanwhile, 16.39% of genes have introns, and 83.60% genes were intron-less. Further, in *G. arboretum*, 80 *DREB* genes were also divided into three groups, among which, I, II, and III group contained 24, 23, and 33 genes, respectively (Fig. 5C). In group I, motif 1, 2, and 3 were observed. In group II, motif 2 and 4 were conserved. In group III, motif 1 and 2 were conserved. For *DREB* genes in *G. arboretum*, all genes do not contain UTR. Meanwhile, 11.25% of genes contained introns, and 88.75% genes have no introns. Finally, the evolutionary tree classified 79 *DREB* genes of *G. raimondii* into group I (34), II (23), and III (22) (Fig. 5D). In group I, motif 1 and 3 were conserved. In group II, motif 1 was conserved. In group III, motif 1 and 2 were conserved. For *DREB* genes in *G. raimondii*, 32.91% of genes were UTR-less, and both UTR and CDS were detected in 67.08% of genes. Meanwhile, 17.72% of genes contain introns, and 82.27% of genes have no introns.

### Subcellular localization prediction of DREB proteins in cotton

**Fig. 6:**
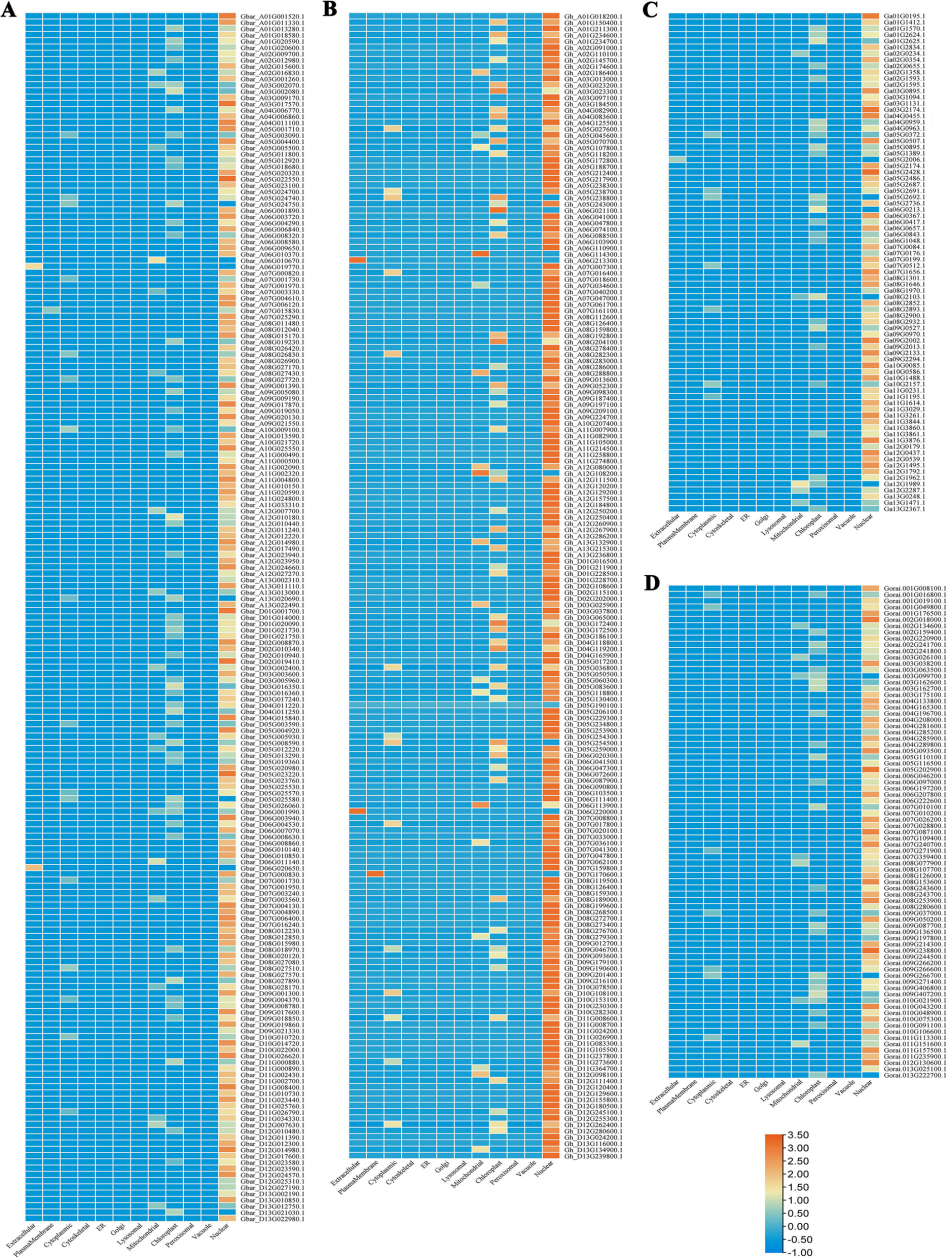
Heat map of the subcellular localizations of DREB proteins. Subcellular localization of DREB proteins were predicted in **A** *G. barbadense*, **B** *G. hirsutum*, **C **G. arboreum, and **D** *G. raimondii*. The color scale represented the abundance of DREB proteins in the cellular components

Subsequently, the subcellular localization of DREB proteins in *G. barbadense*, *G. hirsutum*, *G. arboreum*, and *G. raimondii* were predicted, respectively, by two bioinformatics analysis. As expected, we confirmed that as indicated in heat map, 90.15% DREB proteins of *G. barbadense* were predicted in nucleus, and 6.73% proteins were found in chloroplast (Fig. 6A). The 1, 2, and 3 DREB proteins were found in cytoplasmic, extracellular, and mitochondrial spaces, respectively (Fig. 6A). 89.61% DREB proteins of *G. hirsutum* were located primarily on nucleus, and 6.55% were located on chloroplast (Fig. 6B). Only 1, 2, and 4 DREB proteins were predicted in plasma membrane, extracellular, and mitochondrial spaces, respectively (Fig. 6B). In 80 DREB proteins of *G. arboretum*, 86.25% proteins were predicted on nucleus, and 8.75% were predicted on chloroplast (Fig. 6C). Only 2, 1, and 1 protein were found, respectively, in cytoplasmic, extracellular, and mitochondrial spaces (Fig. 6C). In *G. raimondii*, 88.60% DREB proteins were located on nucleus, and 8 and 1 proteins were predicted on chloroplast and mitochondrial spaces, respectively (Fig. 6D).

### The expression of cotton *DREB* family genes under osmotic and salt stress

**Fig. 7:**
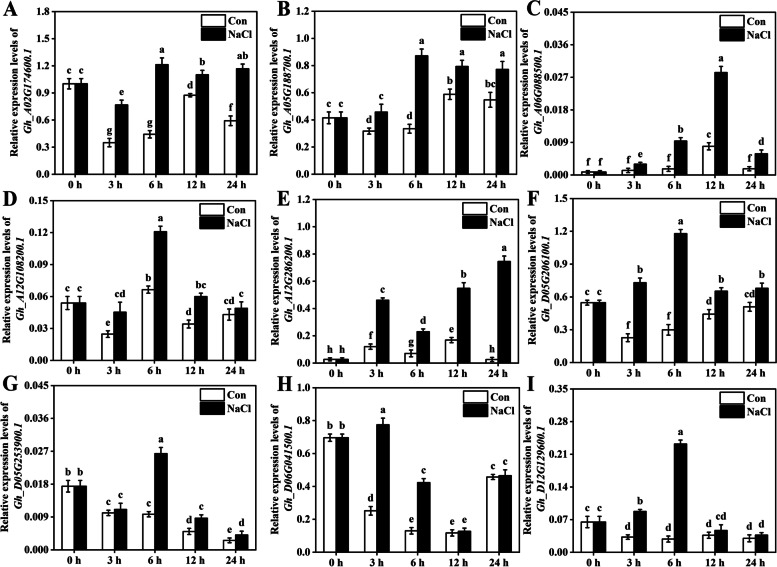
The expression profile of *DREB* genes in the leaves of cotton in response to NaCl stress. **A**-**I** Two-week-old cotton seedlings were treated with 200 mM NaCl (or untreated, as a control) for 0, 3, 6, 12, and 24 h. Next, the relative expression of *DREB* genes was determined by qPCR, respectively. *Gh_A02G174600.1* expression in the control group (Con) at 0 h was set to 100%

**Fig. 8:**
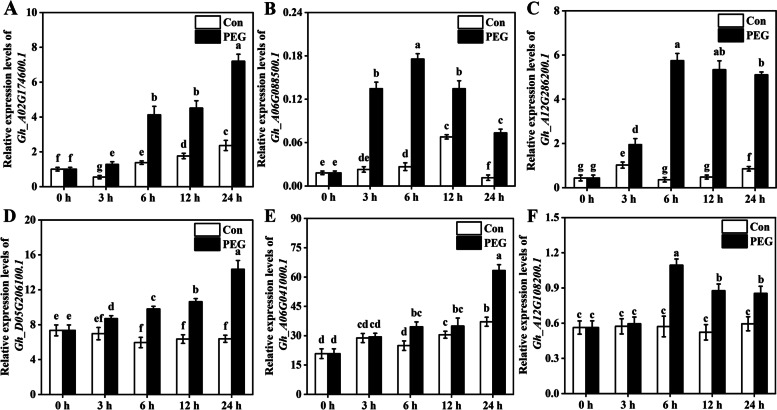
Time-course of changes in the expression of cotton *DREB* genes under PEG treatment. **A**-**F** Two-week-old cotton seedlings were treated with 20% PEG (or untreated, as a control) for 0, 3, 6, 12, and 24 h. Next, the transcription profiling of *DREB* genes was determined by qPCR, respectively. *Gh_A02G174600.1* expression in the control group (Con) at 0 h was set to 100%

To examine the changes in *DREB* gene expression of cotton in response to early osmotic and salt stress, the cotton (*G. hirsutum*; TM-1) seedlings were subjected to NaCl and PEG treatment, respectively. In our experimental conditions, the expression levels of *DREB* family genes, such as *Gh_A02G174600.1*, *Gh_A05G188700.1*, *Gh_A06G088500.1*, *Gh_A12G108200.1*, *Gh_A12G286200.1*, *Gh_D05G206100.1*, *Gh_D05G253900.1*, *Gh_D06G041500.1*, and *Gh_D12G129600.1*, were examined by qPCR in leaves of cotton seedlings under NaCl treatment. As expected, our results showed that in compare with normal conditions, the expression of above *DREB* genes were differentially induced by salt stress (Fig. 7). Similarly, the expressions of *Gh_A02G174600.1*, *Gh_A06G088500.1*, *Gh_A12G286200.1*, *Gh_D05G206100.1*, *Gh_A06G041000.1*, and *Gh_A12G108200.1* were evaluated in leaves of cotton seedlings under PEG treatment. As shown in Fig. 8, the induction of *DREB* gene expression were observed under PEG treatment, compared with normal conditions. However, the expression levels of *DREB* genes were differently upregulated during osmotic and salt stress at 0, 3, 6, 12, or 24 h. Collectively, above results confirmed that the transcript abundances of *DREB* genes in cotton were increased under early osmotic and salt stress.

## Discussion

Ample reports suggested that *DREB* subfamily was a class of important members of *AP2/ERF*s family in plants [10, 11], which were widely distributed in plants [21, 38–41]. It is demonstrated that *DREB* family genes in plants function the vital roles by either directly responding to environmental stresses or regulating the expression of downstream target genes [13, 14]. Cotton is the important economic crops providing the natural textile fiber and the amount of edible oil worldwide [31]. However, the knowledge of *DREB* gene family in cotton is limited.

To fill this gap, we identified 193, 183, 80, and 79 *DREB* family genes in *G. barbadense*, *G. hirsutum*, *G. arboreum*, and *G. raimondii*, respectively (Fig. 1A). These 535 *DREB* genes from four cotton species were analyzed in this study. Based on the subgroup(s) categories of *Arabidopsis thaliana*, 535 *DREB* family genes from four cotton species were divided into 6 subgroups (Fig. 1), which was similar to those in other species [12, 21, 38, 39]. These results above indicated that *DREB* family genes were widespread in plants. Herein, the chromosomal location showed that *DREB* family genes from four cotton species were unevenly distributed on their respective chromosomes (Fig. 2), and these partly showed the evolutionary information of cotton *DREB* family. It is reported that the asymmetric arrangement of genes on chromosomes could show the information of their evolution [39].

The gene duplication, such as whole genome duplications (WGD), segmental duplications, and tandem duplications, was also major forces of the genome evolution in plants, and could alter the genetic system by generating the new gene subfamilies [42]. In our study, the possible WGD, segmental duplication, and/or tandem duplication events in *DREB* gene family were investigated, respectively, in four cotton species (Figs. 3 and 4). As expected, our results showed that in each cotton species, WGD, segmental duplication, and/or tandem duplication events were confirmed to occur during the evolution of cotton *DREB* gene families (Fig. 3). Meanwhile, we observed that the rates of WGD or segmental duplication were higher than tandem duplication events, suggesting that WGD or segmental duplication might mainly be driving forces of evolution for *DREB* gene families. These results above suggested that *DREB* gene families were expanded mainly by WGD or segmental duplications (in particular) and tandem duplications, and this might have enhanced the wide adaptability of cotton. Similarly, WGD, segmental duplications, and/or tandem duplications have been identified during the evolution of the *S. spontaneum*, *B. campestris*, and soybean [20, 43, 44]. It is known that the tandem duplications play the adaptively important roles in the evolution of abiotic stress responsive genes. Previously, a report confirmed that the tandem repeats contained the common *cis*-acting elements, and might play similar functions [45]. Hence, our results showed that tandem duplication pairs of *DREB* genes might preform the similar functions, and the common *cis*-acting regulatory elements were identified in promoter region of *DREB* genes (Fig. 5). To further explore the underlying evolutionary mechanisms of *DREB* gene family, the syntenic relationships among four cotton species were was inferred (Fig. 4). Our results demonstrated that a lot of collinearity gene pairs were observed between *G. hirsutum* and *G. barbadense*, *G. arboreum*, or *G. raimondii*. These might be explained by the closer phylogenetic relationships between diploid species and tetraploid species. Due to the most plants were diploidized polyploids, the segmental duplications were often found in plants [46]. WGD or polyploidy, is also the significantly source of evolution, resulting in new traits and transcriptional regulatory sites that alter gene expression patterns [47]. Our results not only confirmed that during the evolution of cotton, *DREB* gene family occurred the gene duplication and polyploidization (Figs. 3 and 4), but revealed that the evolutionary analysis of *DREB* gene family based on the tetraploid nature of cotton could offer a reference to investigate the evolutionary tend of other genes.

To identify the evolutionary relationship of *DREB* gene family, the arrangement of gene structures and conserved motifs were analyzed respectively in *G. barbadense*, *G. hirsutum*, *G. arboreum*, and *G. raimondii*. Firstly, for each cotton species, all *DREB* genes were divided into three subgroups (I-III) based on their evolutionary relationship (Fig. 5). Next, one conserved motif (motif 1) in all *DREB* genes contained the conserved AP2 domain (Additional file 3) were identified (Fig. 5), suggesting that it was conserved highly during the evolution of cotton. Additionally, for each cotton species, the conserved motifs were similar in the same subgroup (Fig. 5). Notedly, we observed that in *DREB* genes located on the same chromosome, some conserved motifs were presented. The similar results have been showed in the previous studies [48]. These results above might be attributed to the polyploid changes that occurred in plant genome. Analysis of the conserved domains showed that the conserved motifs of *DREB* gene family from four cottons were present in almost all genes (Fig. 5), which indicated that the domains of *DREB* family genes were highly conserved. However, the similarity of other sequences was lower compared to the domains, which indicated that *DREB* family genes of cotton have shown some diversity during the evolution process. Similar to these results, the distribution of gene structure and conserved motifs provided an insight to evolutionary relationship [49]. The promoter *cis*-acting elements were involved in the regulation of gene expression, and therefore the gene function could be preliminarily predicted by analyzing the promoter *cis*-acting elements of *DREB* family genes. Further, the *cis*-acting elements of *DREB* family genes were predicted in four cottons, respectively (Fig. 5). These results suggested that expect the basic elements TATA-box and CAAT-box, the *cis*-acting elements of *DREB* family gene could be divided into three categories: plant growth and development elements, phytohormone- response elements, and stress-response elements (Fig. 5). Moreover, we found that the promoter regions of *DREB* genes contained more elements related to light response (Fig. 5), which indicated that *DREB* family genes might have an intrinsic relationship with circadian rhythm regulation. Additionally, MeJA responsive elements, salicylic acid responsive elements, and defense and stress responsive elements were identified in all four species (Fig. 5), suggesting that *DREB* family genes might play important role in biotic stress resistance. Similarly, *AP2/ERF-DREB* family genes were identified as having a role in biotic stress responses in potato [50]. The structural analysis of *DREB* family genes from four cottons showed that most *DREB* genes (76.68–88.75%) did not contained introns (Fig. 5). And, the proportion of UTR and CDS showed significantly different in each cotton species. The diversity of *DREB* gene structure could elucidate the functional diversification, which might be due to evolutionary changes in plant genome [51].

Generally, TFs are involved in diverse plant processes via specifically binding to *cis*-elements in the promoter region of target genes [30], and thus one of the important features of TFs is its nuclear localization. In this study, the subcellular localization of DREB proteins was predicted in four cotton species based on bioinformatics analysis, suggesting that DREB proteins might be primarily located in the nucleus (Fig. 6). However, these results should be checked by using the fluorescent proteins tag-based imaging techniques in near future. Our results were consistent with the previous study [39], and supported by that *DREB* transcription factors play mainly the important roles in regulation of gene expression [19].

*G. hirsutum* (cotton TM-1), which is widely used as a genetic standard line, is cultivated extensively, accounting for more than 90% of cultivated cotton worldwide [34, 37]. A large number of genes and gene families have been studied in *G. hirsutum*. So, it was used for gene expression analysis in our study. Additionally, *DREB* genes for analyzing the salt/osmotic stress responses were picked based on the gene expression analysis from Cotton Functional Genomics Database, in which the expression of these genes was significantly induced under stress condition. Herein, these *DREB* genes were further confirmed as candidate genes involved in response to early salt (Fig. 7) and osmotic (Fig. 8) stress in *G. hirsutum*. However, *GhDREB* genes showed different expression levels during salinity and osmotic condition (Figs. 7 and 8), implying the functional divergence of *GhDREB* family members. This is consistent with the above conclusion that the different *cis*-acting elements were detected in same *DREB* subfamily (Fig. 5). Similarly, the overexpression of *TaDREB3* could increase the tolerance of wheat to high temperature, dehydration, and salt stresses [24]. In barley, *DREB* gene expression was upregulated in response to salt and drought stress [23]. The expression of *DvDREB2A* in chrysanthemum could be induced by drought, high salt, and low temperature [52]. Collectively, the previous studies suggested that in plant kingdom, many *DREB* genes were involved in response to multiple abiotic stresses, such as salt, extreme temperature, and drought stimuli [10, 20, 52, 53].

Taken together, we have identified *DREB* subfamily genes from four cotton species, including *G. barbadense*, *G. hirsutum*, *G. arboreum*, and *G. raimondii*. Our results might facilitate future research on *DREB* subfamily genes in cotton species, and provide a foundation to further explore the underlying roles of this important transcriptional factor family. These results may have applications in the selection of valuable candidate *DREB* family genes for functional studies, and shed light on the potential roles of genetic improvement involved in agricultural production and stress tolerance in cotton crops.

## Conclusions

In this study, we addressed the 535 *DREB* family genes of *Gossypium barbadense*, *Gossypium hirsutum*, *Gossypium arboreum*, and *Gossypium raimondii*. Phylogenetic analysis confirmed that all *DREB* genes were classified into six groups. Further, the evolutionary relationship and diversification of cotton *DREB* genes were systematic investigated, among which, the phylogeny, chromosomal distribution, synteny analysis, gene structure, subcellular localization, and gene expression in response to salinity and osmotic stress were carried out on each cotton species. These findings from this study will provide valuable information for investigating cotton *DREB* genes, and lay the foundation for functional research of *DREB* genes in near future.

## Methods

### Identification of the *DREB* gene family in four cotton species

The gene sequences of *Arabidopsis AtDREB* were retrieved from the Plant Transcription Factor Database (PlantTFDB; http://planttfdb.gao-lab.org). The genome sequences of *Gossypium barbadense* (AD2), *Gossypium hirsutum* (AD1), *Gossypium arboreum* (A2), and *Gossypium raimondii* (D5) were obtained from Cotton Functional Genomics Database (https://cottonfgd.org/ [54]). Based on the unique domain of the *DREB* family genes, AP2 domain (PF00847), Hidden Markov Model (HMM) profile of the PF00847 were obtained from Pfam 35.0 (http://pfam.xfam.org/). All coding domain sequences (CDS) contained PF00847 in four cotton species were searched by HMM search function of ToolKit Biologists Tools (TBtools) software (https://github.com/CJ-Chen/TBtools) to obtain possible *DREB* family members. The amino acid sequences were searched for conserved domains using CDD-Search in NCBI and SMART software, and the members of *DREB* family members were identified according to the number and type of conserved domains.

### Construction of phylogenetic tree of cotton *DREB* family genes

The phylogenetic trees of *DREB* subfamily genes from four cottons were constructed by MEGA (V7.0) software. The multiple sequence alignment of protein sequences for *DREB* genes coding region in *Arabidopsis* and four cottons (*G. barbadense*, *G. hirsutum*, *G. arboreum*, and *G. raimondii*) were carried out by ClustalW tool with default parameters [55]. The best model of phylogenetic tree was calculated and constructed with MEGA, and then a phylogenetic tree of all DREB proteins was constructed using the maximum likelihood method with the following parameters: poisson correction, pairwise deletion, and 1000 bootstrap replicate. The constructed evolutionary tree was visualized and beautified with the online iTOL tool (https://itol.embl.de/).

### The physical distribution of cotton *DREB* family genes on the chromosome

According to the initial position and length of chromosome, MapChart software (https://www.wur.nl/en/show/Mapchart.htm [56]) was employed to generate the distribution of cotton *DREB* genes on their corresponding chromosomes in silico. The chromosome location information and genome annotation files of *DREB* family genes in *G. barbadense*, *G. hirsutum*, *G. arboreum*, and *G. raimondii* are obtained from the cotton functional genomics database. Chromosome distribution of *DREB*s genes from the four cottons was presented by using TBtools software.

### Gene duplication and micro‑synteny analysis

The collinearity and the syntenic relationship were investigated among *G. barbadense*, *G. hirsutum*, *G. arboreum*, and *G. raimondii*. The complete genomic sequences of above four cotton species as well as respective genome annotation files were analyzed by using the Multiple collinear scanning toolkits (MCScanX [57]) of TBtools software. The obtained results were subjected to TBtools software. The analysis of gene duplication events and the syntenic relationship between the *DREB* genes among four cotton species were carried out by MCScanX tool [57] and Dual Synteny Plotter software (https://github.com/CJ-Chen/TBtools/) [58], respectively. Advanced circos was used to visualize the collinearity of homologous genes based on the homology between each species and their positions on the genome [58]. Circos Plot and synteny images were constructed and visualized through advanced Circos and dual synteny plotter software to examine segmental duplication gene pairs and orthologous gene conservation of *DREB* genes.

### Analysis of conserved motif, promoter *cis*-acting elements, and gene structure of *DREB* family genes

MEME (Multiple Em for Motif Elicitation) tool (5.4.1 version, https://meme-suite.org/meme/tools/meme) [59] was employed for the analysis of conserved motifs from DREB proteins in *G. barbadense*, *G. hirsutum*, *G. arboreum*, and *G. raimondii*. The phylogenetic trees and conserved motifs were integrated by using TBtools software.

The promoter sequences (2.0 kb of sequence upstream from the transcription start site) of *DREB* genes of four cottons were obtained from the cotton genomics database. The promoter *cis*-acting elements of *DREB* genes are predicted by the PlantCARE online website (http://bioinformatics.psb.ugent.be/webtools/plantcare/html/), and visualized through the TBtools software.

The gene structure information and genome annotation files of *DREB* subfamily genes in *G. barbadense*, *G. hirsutum*, *G. arboreum*, and *G. raimondii* were obtained from the cotton functional genomics database. The information of conserved domains was obtained by the CDD-Search function of NCBI. The merging and mapping of these results were conducted by the TBtools software. The exon/intron structure of each *DREB* gene was analyzed by using the online Gene Structure Display Server 2.0 (http://gsds.cbi.pku.edu.cn/) [60].

### Subcellular localization prediction of DREB proteins

The subcellular localizations of all DREB proteins of *G. barbadense*, *G. hirsutum*, *G. arboreum*, and *G. raimondii* were predicted by WoLF PSORT online tool (https://wolfpsort.hgc.jp/) [61] and CELLO Web server (http://cello.life.nctu.edu.tw/) [62]. The heat maps of these results were conducted by TBtools software.

### Plant materials and growth conditions

The cotton seeds (*G. hirsutum*; TM-1) was provided by Dr. Fuguang Li (Chinese Academy of Agricultural Sciences). Cotton seeds with full particles were selected and soaked in 30% hydrogen peroxide for 1 h to soften the seed coat. Afterwards, the seeds were washed with ultrapure water for 3–5 times to remove the residual hydrogen peroxide. The washed cotton seeds were placed overnight in a petri dish containing distilled water before sowing. Plants were grown for 14 days under the following conditions: 16/8 h (25/26 °C) light/dark cycle in growth chamber [60].

For analysis of osmotic stress tolerance, the two-week-old cotton seedlings were treated with or without 20% PEG6000 for 0, 3, 6, 12, and 24 h, respectively. In order to assay the salt stress tolerance, the two-week-old cotton seedlings were treated with and without 200 mM NaCl for 0, 3, 6, 12, and 24 h. The leaves of cotton seedlings harvested at the indicated time points, were immediately used or frozen in liquid nitrogen, and stored at − 80 °C until further determination.

### RNA extraction and real-time quantitative PCR (qPCR)

According to pervious method, the total RNA of cotton leaves was extracted with the Plant total RNA extraction kit (DP441; Tiangen, Beijing, China). RNA concentration was evaluated using the NanoDrop 2000 (Thermo Fisher, Wilmington, USA), and the quality was detected by 1% agarose gel electrophoresis. cDNAs were then synthesized with HiScript®IIQ RT SuperMix for qPCR (Vazyme, Nanjing, China).

According to the gene-specific primers (Additional file 4), the expression of *DREB* family genes in leaves of PEG- and NaCl-treated cotton seedlings was conducted using the Roche Light Cycler®96 automatic fluorescence quantitative PCR instrument (Roche, America) with ChamQ Universal SYBR qPCR Master Mix (Vazyme, Nanjing, China). Three biological and three technological repeats were performed in qPCR. Relative expression levels of *DREB* genes were calculated by using the 2^−ΔΔC^_T_ method [63], and were presented as values relative to that of corresponding control samples at the indicated times. The constitutively expressed *Actin7* gene was used as the internal standard control.

### Statistical analysis

The statistical analyses were performed using SPSS software (16.0 version; SPSS Inc., Chicago, IL, USA). Data are expressed as the mean ± SE from three independent experiments with three biological replicates for each. According to Duncan's multiple range test, differences were assessed using one-way analysis of variance (ANOVA) at *P* < 0.05.

## Supplementary Information

**Additional file 1:** **Table S1.** WGD or segmental and tandem gene pairs.

**Additional file 2:** **Table S2.** List of syntenic gene pairs.

**Additional file 3:** **Fig. S1.** The phylogenetic relationships and AP2 domains in **A** *G. barbadense*, **B** *G. hirsutum*, **C** *G. arboretum*, and **D ***G. raimondii*.

**Additional file 4:** **Table S3.** The sequences of primers for qPCR.

## Data Availability

The datasets supporting our conclusions of the current study are included in the manuscript and additional file. The *Arabidopsis* DREB protein sequences were downloaded from the Arabidopsis information source database (http://www.arabidopsis.org). We did not generate any sequencing data in this study. We have used already published genomic data. The genomic data of cotton were obtained from the Cotton Functional Genomics Database (https://cottonfgd.org/). The raw sequence data of *G. barbadense* are available in NCBI-BioProject database under accession number PRJNA433615. Data from *G. hirsutum* were retrieved from NCBI-BioProject database under accession number PRJNA503326. The genomic data of *G. arboreum* were accessible through NCBI-BioProject PRJNA382310. Similarly, data of *G. raimondii* were obtained from NCBI-Bioproject PRJNA171262.
