# Supplementary material for: Genome-wide identification and expression analysis of DREB family genes in cotton
Source: BMC Plant Biol. 2023 Mar 30;23:169. doi: 10.1186/s12870-023-04180-4 (PMC10061749; doi:10.1186/s12870-023-04180-4)
Supplement: Supplementary file 3 — Additional file 3: Fig. S1. The phylogenetic relationships and AP2 domains in A G. barbadense, B G. hirsutum, C G. arboretum, and D G. raimondii. [file 12870_2023_4180_MOESM3_ESM.doc]

**Fig. S1**



**Fig. S1** The phylogenetic relationships and AP2 domains in *G. barbadense* **A**, *G. hirsutum* **B**, *G. arboretum* **C**, and *G. raimondii* **D**.
