# Supplementary material for: Genome-wide identification and expression analysis of DREB family genes in cotton
Source: BMC Plant Biol. 2023 Mar 30;23:169. doi: 10.1186/s12870-023-04180-4 (PMC10061749; doi:10.1186/s12870-023-04180-4)
Supplement: Supplementary file 4 — Additional file 4: Table S3. The sequences of primers for qPCR. [file 12870_2023_4180_MOESM4_ESM.doc]

**Table S3.** The sequences of primers for qPCR

| Primer | Primer Sequence 5’ to 3’ |
| --- | --- |
| *Gh_A02G174600.1* | F: GCTCCCCATTGAACGGTAGT  R: GGGGTGAGTTCGTTAAGCCC |
| *Gh_A06G088500.1* | F: ATCCAATCCGAGCAATCCAAC  R: ACCCGTCCATAGGCAACTCAC |
| *Gh_A12G286200.1* | F: GCACTGAGAGGGAAGTCAGC  R: CTGAAAGTCTCCGCCACCTC |
| *Gh_D12G129600.1* | F: CGAAAGCGGCACATTTAGTAGT  R: GGCGAAAGTCCATCCACAAG |
| *Gh_D06G041500.1* | F: TTCGATACAGCCGAGGAAGC  R: GCCACCGATATGGGAACCTT |
| *Gh_A06G041000.1* | F: CTTAACGCCAACCCCAACAC  R: CCAAAGACGTGTCCGGTTCT |
| *Gh_D05G206100.1* | F: CAGAGAACCCAACAAGCGGT  R: GGAAGGACCACGCAAGTAGA |
| *Gh_A05G212400.1* | F: CATCCAAGGTTACGGCAGACA  R: GATACAGCCGAACCCTCACTCT |
| *Gh_D05G229300.1* | F: TGCCTTCTTCTGTTGACGCT  R: GTGTCTGCCGTAACCTTGGA |
| *Gh_A05G188700.1* | F: GTCCAGCAAGCGGAGAGAAG  R: CCAAATCCTGGACCGCTTGT |
| *Gh_A12G108200.1* | F: AAACTCTACAGGGGAGTGCG  R: AAGCTGAGCCTGGCAAAGTC |
| *Gh_D05G253900.1* | F: CCAAGTCCGTACAGAAAGCGG  R: CCAGGAGCCACCACCACAAC |
| *Action 7* | F: TATGTTGCCATCCAGGCCGTTC |
|  | R: AACACCATCACCGGAATCCAGC |
